# Effects of Somatothermal Far-Infrared Ray on Primary Dysmenorrhea: A Pilot Study

**DOI:** 10.1155/2012/240314

**Published:** 2012-12-18

**Authors:** Yu-Min Ke, Ming-Chiu Ou, Cheng-Kun Ho, Yung-Sheng Lin, Ho-Yen Liu, Wen-An Chang

**Affiliations:** ^1^Department of Obstetrics and Gynecology, Taichung Veterans General Hospital, Taichung 40705, Taiwan; ^2^Department of Applied Cosmetology and Master Program of Cosmetic Science, Hungkuang University, Taichung 43302, Taiwan

## Abstract

The purpose of this study was to assess the beneficial effects of using a far-infrared (FIR) belt on the management of patients with primary dysmenorrhea. This is the first study to determine the efficacy of somatothermal FIR using a parallel-arm randomized sham-controlled and double-blinded design with objective physical evidence and psychometric self-reports. Fifty-one Taiwanese women with primary dysmenorrhea were enrolled in the study. Results indicate that there was an increased abdominal temperature of 0.6°C and a 3.27% increase in abdominal blood flow in the FIR group (wearing FIR belt) compared to those in the control group (wearing sham belt). Verbal rating scale and numeric rating scale scores in the FIR group were both lower than those in the control group. Compared to the blank group (wearing no belt), the average dysmenorrhea pain duration of the FIR group was significantly reduced from 2.5 to 1.8 days, but there was no significant difference in the control group. These results demonstrate that the use of a belt made of far-infrared ceramic materials can reduce primary dysmenorrhea.

## 1. Introduction

Dysmenorrhea is pain with menstruation, which may be accompanied by headache, dizziness, nausea, vomiting, diarrhea, cold sweats, or other symptoms [1, 2]. For clinical purposes, dysmenorrhea is classified into primary and secondary dysmenorrhea. Previous studies showed that 50% to 80% of women worldwide have experienced dysmenorrhea, and the majority of these women are teenagers and have primary dysmenorrhea [2, 3]. Primary dysmenorrhea, a condition associated with ovulatory cycles, is the occurrence of menstrual cramps in the absence of demonstrable disease that could account for symptoms [4]. After the onset of menstruation, pain typically starts within hours peaking between 12 and 24 hours and persisting for 12–72 hours.

Clinical research has shown that the main cause of primary dysmenorrhea may be associated with increased production of endometrial prostaglandin, resulting in a high concentration of prostaglandins in blood. The pain-related biomolecular induction of cyclooxygenase (COX-2) and prostaglandin is strongly associated with the severity of primary dysmenorrhea [4]. Excessive prostaglandin causes uterine contractions, ischemia, cramping, and pelvic pain. Nonsteroidal anti-inflammatory drugs (NSAIDs) are commonly used in women with dysmenorrhea, because they can inhibit prostaglandin synthesis and effectively alleviate the symptoms of dysmenorrhea. However, NSAIDs may lead to many adverse effects, including indigestion, headache, and drowsiness, and their rate of failure to reduce dysmenorrhea may reach 20% [5]. Therefore, complementary and alternative medicine (CAM) is becoming an increasingly popular choice for alleviation of dysmenorrhea [6–13].

For women with dysmenorrhea, the application of local heat can reduce muscle tension and relax abdominal muscles to reduce pain caused by muscle spasms. Heat can increase pelvic blood circulation to eliminate local blood and body fluid retention and diminish congestion and swelling, thereby enabling a reduction in pain caused by nerve compression. Previous studies reported that local heat inhibits pain signals and increases proprioception [14, 15].

Heating pads and hot water have long been used as a folk remedy for the treatment of dysmenorrhea [14]. Akin et al. [14] reported that a heating patch on the abdomen can be as effective as ibuprofen to treat dysmenorrhea. However, external energy is needed to provide the heat, and there is a risk of burn accident with improper temperature control. In addition, there are a number of disadvantages using complementary and alternative medicine therapies, such as Chinese herbal medicine [12], acupuncture [16], acupressure [7, 17], aromatherapy [8], and transcutaneous electrical nerve stimulation [11], which may include inconvenience of application and energy consumption, among others.

Far-infrared (FIR) rays are invisible electromagnetic waves, which can produce many biological effects including thermal and nonthermal effects [18–23]. When FIR materials are used to cover the skin, it can reduce body heat loss owing to their thermal insulation properties [24–26]. FIR can promote microcirculation, accelerate wound healing, modulate sleep, and treat depression [27–29]. In general, as an alternative nonpharmacologic therapy, FIR has been shown to be an effective and safe modality for promoting health in patients with various medical conditions.

The use of electrothermal FIR belts at a temperature of 50°C was recently reported to be an effective treatment for primary dysmenorrhea [30, 31]. The most common adverse event was burn caused by the incorrect use of the hot pack [30]. While a number of studies have investigated the therapeutic effects of electrothermal FIR belts, to our knowledge the health benefits of somatothermal FIR belts have not been previously reported in the literature. Thus, the aim of the present study was to test the effectiveness of a somatothermal FIR belt as a novel alternative therapy for the relief of primary dysmenorrhea. 

## 2. Materials and Methods

### 2.1. Participants

The clinical trial was approved by the institutional review board (IRB) at HungKuang University, (Project number 98-B-002). The participants were screened by a gynecologist. Inclusion criteria were age 18 years or older which can legally give consent to participate in this clinical trial and history of painful menstruation for at least one year. Physical examination and ultrasonography were used to support diagnosis of primary dysmenorrhea. Patients who had gynecological diseases or who used other FIR products within one month before the study were excluded. Patients were instructed not to use any type of medication, such as NSAIDs or oral pills, to relieve painful menstruation during the study period. In the last analysis, a total of 51 subjects aged 19 to 35 years were enrolled in this study. The investigation was conducted between January 1, 2010 and December 31, 2010.

### 2.2. Belts

Two kinds of belts each measuring 15 cm × 70 cm were used in this study. The FIR belt and the control belt were embedded with and without 10 wt% FIR ceramic powders, respectively. As previously reported [19–23], the FIR ceramic powders were composed of numerous mineral oxides, including aluminum oxide, ferric oxide, magnesium oxide, and calcium carbonate. The FIR energy, at wavelengths between 3 and 16 *μ*m, was 10.16 mW/cm^2^ as determined using an SR5000 spectroradiometer (CI, Ltd., Migdal HaEmek, Israel) at the Industrial Technology Research Institute, Taiwan. 

### 2.3. Abdominal Temperature and Blood Flow

All measurements and procedures in this study were performed in a climate-controlled room at a constant temperature and humidity (23°C and 60% relative humidity) [32]. Before assessment, participants were required to lie down in the aforementioned room for 30 minutes to acclimatize to the indoor climatic conditions. Participants were thermographed to measure abdominal temperatures using a Fluke Ti25 thermal imager (Fluke Corporation, Everett, WA, USA), and their abdominal blood flows were detected using a MoorLDI2-IR Laser Doppler Imager (Moor Instruments Ltd., Devon, UK) as described in the previous report [31]. Subsequently, a double-blind method was used to randomly assign subjects to the FIR belt (*n* = 26) group or control belt (*n* = 25) group. Each subject then wore the belt around the abdominal region for 30 minutes. After removing the belt, posttreatment measurements of abdominal temperatures and blood flows were performed. 

### 2.4. Menstrual Pain Assessment

**Figure 1:**
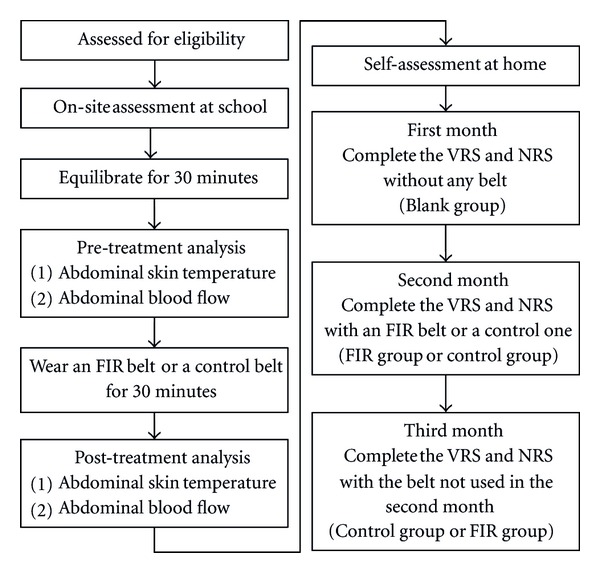
The flow chart of participants through the trial.

All participants (*n* = 51) completed two questionnaires, the verbal rating scale (VRS) and the numeric rating scale (NRS) [33, 34], to assess the extent of pain on days 1 to 3 of three menstrual cycles. As shown in Figure 1, the pain assessment for the first menstrual cycle was conducted without any belt (blank group). For the second and third menstrual cycles, each participant was instructed to wear the belt on the abdominal region for the whole day during menstruation, but could remove it in order to bathe. Application of an FIR belt or a control belt in sequence was also determined by random sampling (double-blind). The six precisely worded descriptions of pain degrees in the VRS (Chinese version) are scored as follows: none (0), very mild (1), mild (2), moderate (3), severe (4), and very severe (5). The 11-point NRS (Chinese version) is scored using eleven levels of pain, ranging from no pain (0) to the worst pain (10). For assessments of pain levels, there may be individual differences in subjects' feelings and cognition. Thus, this study adopted both the VRS and NRS as complementary indices so that participants' pain levels could be more precisely assessed. 

### 2.5. Statistical Analysis

All data between groups before and after applying belts were analyzed by the Wilcoxon Signed-Rank Test using the SPSS 12.0 software package for statistical analysis. A value of *P* < 0.05 was considered statistically significant (*) and *P* < 0.01 was highly significant (**).

## 3. Results and Discussion

### 3.1. Abdominal Temperature

**Figure 2:**
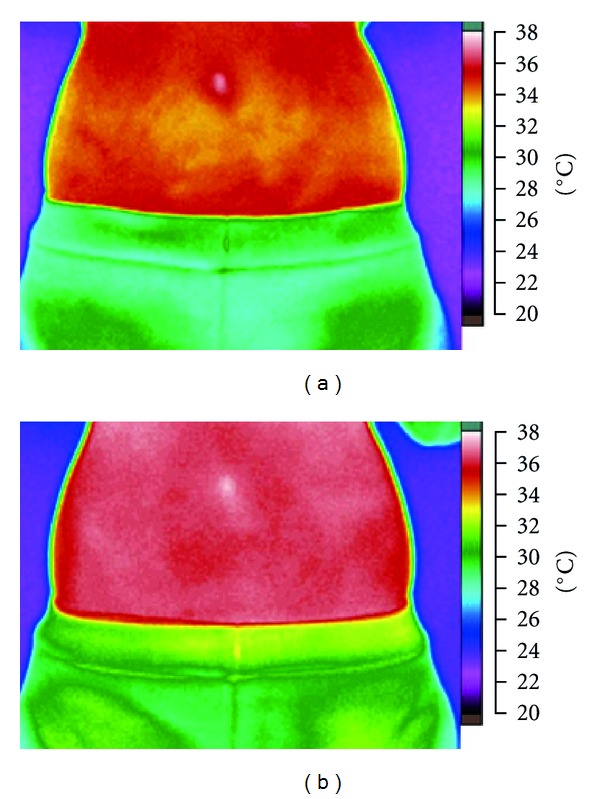
The abdominal skin temperature distribution of pretest (a) and posttest (b) of applying an FIR belt.

**Figure 3:**
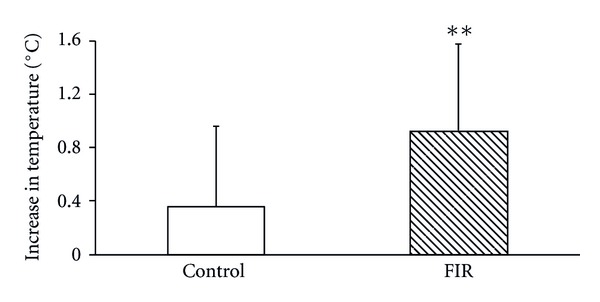
The average increases in temperature after wearing the control belt and FIR belt. The symbol ** indicates a highly significant difference between groups using the Wilcoxon Signed-Rank Test.

Figure 2 shows the results of temperature differences between pretreatment and posttreatment in the FIR belt group. There was a temperature increase after treatment which was probably due to the thermal insulation effect of the belt. The statistical analysis indicates that the average increases in temperature in the control group and FIR group were 0.36°C and 0.93°C, respectively (Figure 3). The temperature increase in the FIR group was thus enhanced by 0.57°C compared with that found in the control group, which demonstrates that the FIR treatment was associated with a greater rise in abdominal temperature than the control condition. It is reasonable to conclude that the thermal insulation properties of the FIR belt resulted in an increase in the subjects' temperatures. This finding is consistent with previously reported results in similar studies [24–26].

### 3.2. Abdominal Blood Flow

**Figure 4:**
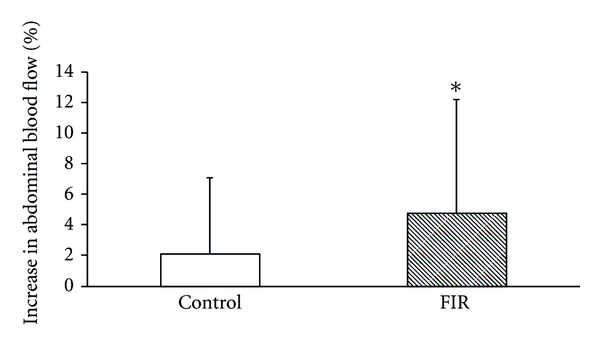
The average increase in abdominal blood flow after wearing the control belt and FIR belt. The symbol * indicates a significant difference between groups using the Wilcoxon Signed-Rank Test.

With the rise in body temperature, there was a corresponding rise in abdominal blood flow after using the belts for 30 minutes. The average blood perfusion units measured by the MoorLDI2-IR Laser Doppler Imager were 116.5 and 119.0 before and after intervention in the control group, and 115.9 and 121.4 in the FIR group, respectively. There were increases in blood flow of 2.12% and 4.78% in the control group and FIR group, respectively (Figure 4). Thus, the increase in abdominal blood flow in the FIR group was enhanced by 2.66% compared with that in the control group.

### 3.3. VRS and NRS Scores

**Figure 5:**
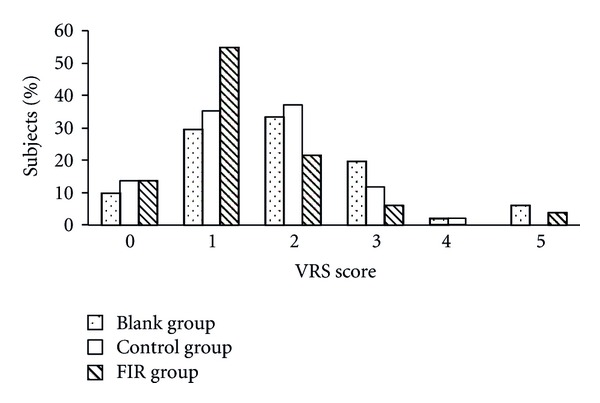
The distribution of subjects' VRS scores on the first day of menstruation.

**Figure 6:**
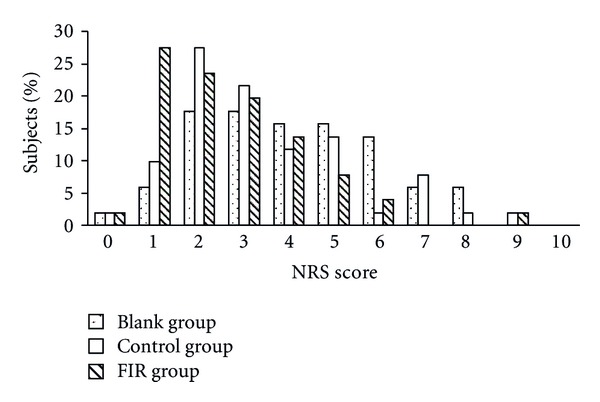
The distribution of subjects' NRS scores on the first day of menstruation.

Figures 5 and 6 show the subjects' distribution of VRS and NRS scores on the first day of menstruation. The results of both VRS and NRS showed a general trend for lower scores in the FIR group. Compared to the blank group, the percentage of subjects with a VRS score above 3 was reduced from 27% to 10% and the percentage of those with an NRS score above 5 was reduced from 41% to 14% in the FIR group. The distribution of scores in the control group was between those of the blank and FIR groups. Therefore, the placebo belt showed some efficacy for pain relief, but a better reduction in pain was reported by patients wearing the FIR belt. 

**Figure 7:**
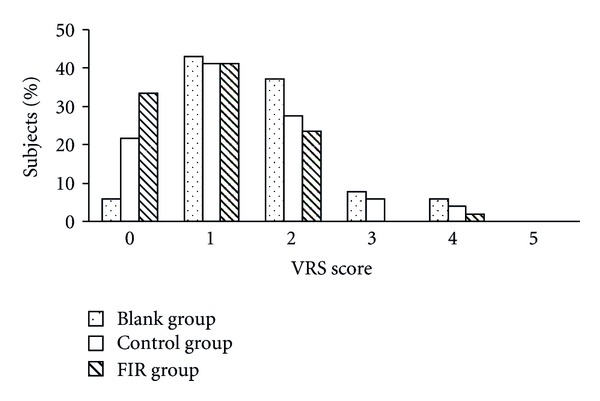
The distribution of subjects' VRS scores on the second day of menstruation.

**Figure 8:**
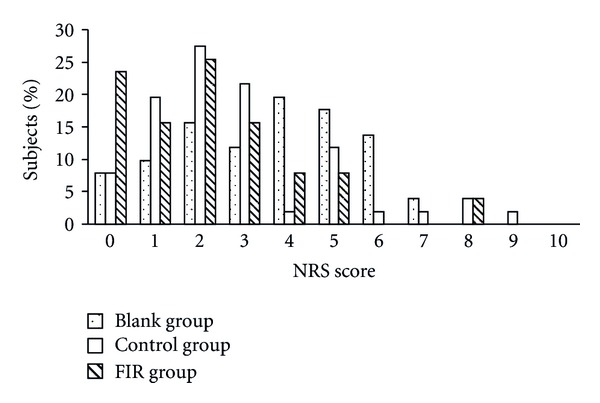
The distribution of subjects' NRS scores on the second day of menstruation.

The results of the second day (Figures 7 and 8) were similar to those found on the first day. After the belt intervention, pain intensity was reduced with respect to baseline. A greater reduction of pain was observed in the FIR belt group as compared with that in the control belt group. Compared with the blank group, the percentage of subjects with a VRS score above 3 was reduced from 14% to 2% (Figure 7) and the percentage of those with an NRS score above 5 was reduced from 35% to 12% (Figure 8) in the FIR group. 

**Figure 9:**
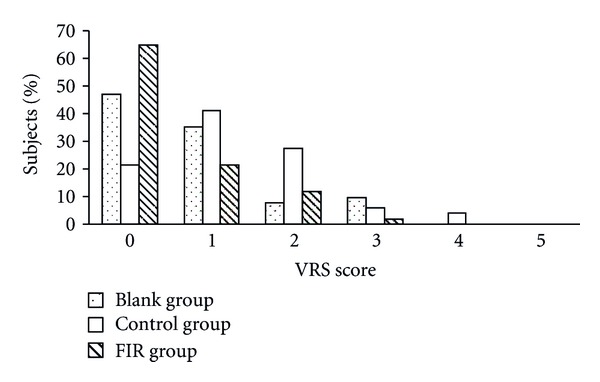
The distribution of subjects' VRS scores on the third day of menstruation.

**Figure 10:**
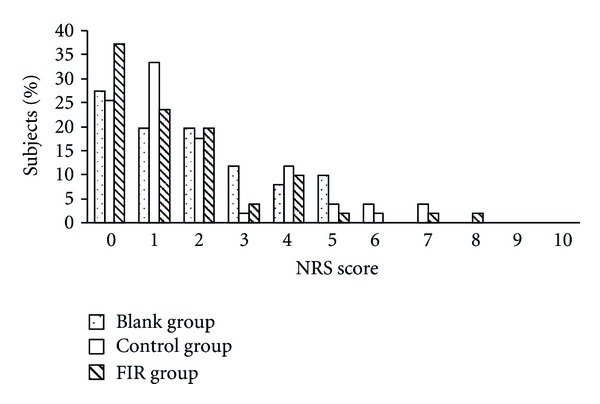
The distribution of subjects' NRS scores on the third day of menstruation.

The results of the third day (Figures 9 and 10) were comparable to the results of the first and second days. The FIR group appeared to be the most efficient in terms of pain reduction. Compared to the blank group, the percentage of subjects with a VRS score above 3 was reduced from 10% to 2% (Figure 9) and the percentage of subjects with an NRS score above 5 was reduced from 14% to 6% (Figure 10) in the FIR group. 

**Figure 11:**
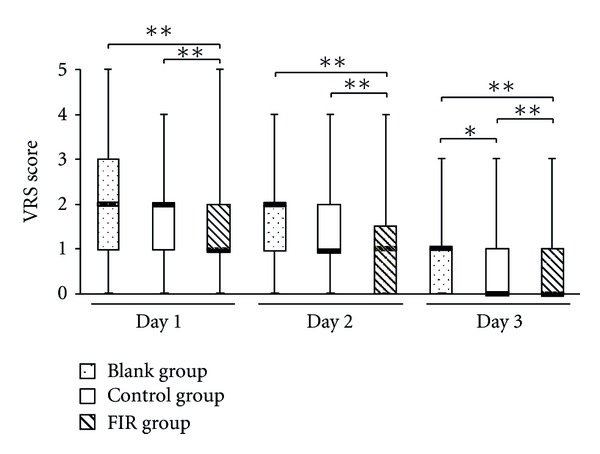
The VRS scores on the first three days of menstruation. The symbols * and ** indicate a significant and highly significant difference between groups using the Wilcoxon Signed-Rank Test, respectively.

**Figure 12:**
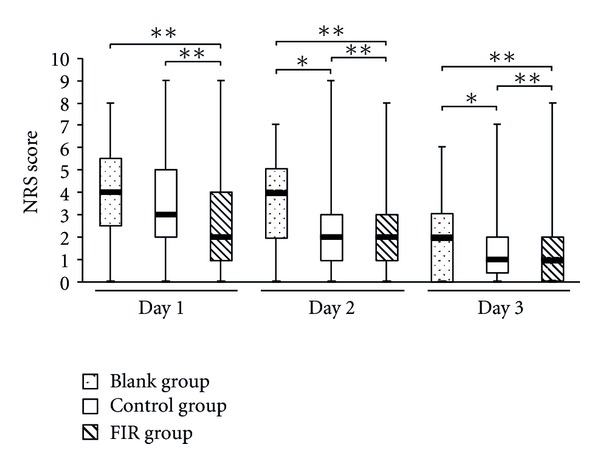
The NRS scores on the first three days of menstruation. The symbols * and ** indicate a significant and highly significant difference between groups using the Wilcoxon Signed-Rank Test, respectively.

**Figure 13:**
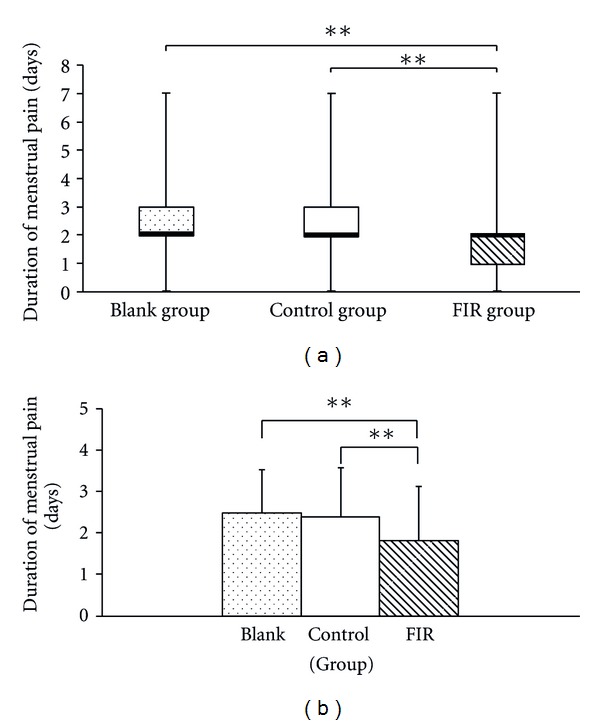
The duration of menstrual pain presented by (a) a box-and-whisker plot and (b) a bar chart indicating the average and standard deviation. The symbol ** indicates a highly significant difference between groups using the Wilcoxon Signed-Rank Test.

Figures 11 and 12 are the box-and-whisker plots of VRS and NRS, respectively. The median levels of VRS and NRS in the FIR group were significantly lower than those of the blank group. The VRS and NRS scores in the FIR group showed highly significant differences compared with those of the control and blank groups. The scores in the control group also showed significant differences compared with those in the blank group on the second day (NRS) and the third day (VRS and NRS).  Figure 13(a) shows the pain duration data and Figure 13(b) indicates their average and standard deviation. The lower and upper quartiles in the FIR group were significantly lower than those in the blank and control groups. Compared to the blank group, it can be seen that duration of pain in the FIR group was significantly decreased in the menstrual period from 2.4 to 1.8 days. However, there was no significant difference between the blank and control groups. Based on these findings, the authors speculate that the application of sufficient warmth to the belly region can improve blood circulation and shorten the menstrual pain duration. 

Previous research has shown that the dysregulation of endometrial blood flow can cause menstrual disorders [35]. Strong and abnormal uterine contractions in women with primary dysmenorrhea can decrease uterine blood flow, resulting in pain in the uterus and the development of ischemia [4]. The application of FIR increases abdominal temperature and blood circulation and reduces muscle tension, leading to the relief of menstrual pain. Related studies also indicate that local heat treatment can alleviate dysmenorrhea [14]. 

In addition to the aforementioned thermal effects, FIR also exerts nonthermal effects in pain relief of dysmenorrhea. Previous studies showed that FIR could increase generation of nitric oxide (NO) and calmodulin in cells [19, 20, 36]. Associated physiological roles of NO include immune regulation [37, 38], neurotransmission [39], and vascular smooth muscle relaxation [40, 41]. Previous reports have demonstrated that NO in endothelial cells plays an important role in regulating smooth muscle [42]. Endothelial NO in smooth muscle cells can activate guanylyl cyclase to produce cyclic guanosine monophosphate (cGMP). The cGMP sequentially activates protein kinase G, reduces smooth muscle intracellular calcium concentration, inhibits myosin light chain phosphorylation, and ultimately promotes smooth muscle relaxation [42–44]. NO has also been shown to help relax the uterus, thereby reducing the degree of dysmenorrhea [45]. 

Ischemia is mainly a consequence of decreased microcirculation or a reduction in local muscle blood flow. This scenario has important consequences for cellular metabolic status, as metabolic and acid-base status is significantly worsened with increased acidosis. During dysmenorrhea, the corresponding regional organic ischemia is associated with increased oxidative stress due to increased levels of reactive oxygen species (ROS), such as superoxide and hydrogen peroxide, which are responsible for destructive processes in organic tissues. We demonstrated that the FIR ceramic material exerted an antioxidant effect by increasing hydrogen peroxide-scavenging activity [21, 22]. 

We also found that FIR induced anti-inflammatory effects by inhibiting prostaglandin E2 (PGE2) [46], and FIR irradiation caused significant inhibition of COX-2 elevation during inflammation [47]. Prostaglandin synthesis is mediated primarily by cyclooxygenase (COX-1 and COX-2), which catalyzes the metabolism of arachidonate to prostaglandin H2 and then produces PGE2 [48], stimulating uterine contractility, which causes the pain. COX-2 and prostaglandin are strongly related to the severity of primary dysmenorrhea. Previous studies also reported that the main cause of primary dysmenorrhea is an abnormal increase in uterine prostaglandin level, leading to pain caused by uterine smooth muscle contraction [49]. Therefore, reducing PGE2 by applying FIR may lessen the discomfort caused by dysmenorrhea. 

## 4. Conclusions

These findings of this study showed that the somatothermal FIR belt was effective as a novel alternative therapy for relief of primary dysmenorrhea. The FIR belt provided better therapeutic effects in terms of pain relief as well as greater elevation of skin temperature and promotion of blood circulation compared with those observed in patients wearing the sham belt. Compared with traditional complementary and alternative medicine therapies for pain reduction in patients with primary dysmenorrhea, our method has a number of advantages, which include convenience and ease of use, and no external energy source is required. We believe that this novel, noninvasive, and convenient FIR therapy may be of practical use in a clinical setting and suggest that further studies be conducted to confirm our results.
